# Preliminary Study of Pet Owner Adherence in Behaviour, Cardiology, Urology, and Oncology Fields

**DOI:** 10.1155/2015/618216

**Published:** 2015-06-22

**Authors:** Zita Talamonti, Chiara Cassis, Paola G. Brambilla, Paola Scarpa, Damiano Stefanello, Simona Cannas, Michela Minero, Clara Palestrini

**Affiliations:** Università degli Studi di Milano, Dipartimento di Scienze Veterinarie e Sanità Pubblica (DIVET), Via Celoria 10, 20133 Milan, Italy

## Abstract

Successful veterinary treatment of animals requires owner adherence with a prescribed treatment plan. The aim of our study was to evaluate and compare the level of adherence of the owners of patients presented for behavioural, cardiological, urological, and oncological problems. At the end of the first examination, each owner completed a questionnaire. Then, the owners were called four times to fill out another questionnaire over the phone. With regard to the first questionnaire, statistically significant data concern behavioral medicine and cardiology. In the first area the owner's worry decreases during the follow-up and the number of owners who would give away the animal increases. In cardiology, owners who think that the pathology harms their animal's quality of life decreased significantly over time. With regard to the 9 additional follow-up questions, in behavioural medicine and urology the owner's discomfort resulting from the animal's pathology significantly decreases over time. Assessment of adherence appears to be an optimal instrument in identifying the positive factors and the difficulties encountered by owners during the application of a treatment protocol.

## 1. Introduction

Owner compliance or adherence to treatment recommendations can determine the success of veterinary treatment [1]. The term compliance describes the observance of a medical recommendation, but it also includes how well laws, regulations, and guidelines are applied when administering prescribed treatments. The concept of compliance in veterinary medicine involves the consistency and accuracy with which a client follows the regime recommended by the veterinarian or other veterinary health care team members [2]. The term “compliance” (or “observance”) suggests that a patient adapts to, submits to, or obeys the instructions of a doctor and implies a submissive role with a professional in a position of authority. The negative connotation of the term has caused the medical world to increasingly distance itself from the term compliance, replacing it with “adherence”; in other words, the tendency to adhere to the doctor's instructions, carrying them out quickly, respectfully, and accurately [3]. The World Health Organization defines adherence as “the extent to which a person's behaviour, taking medication, following a diet, and/or executing lifestyle changes, corresponds with agreed recommendations given by a health care provider” [4]. For these reasons, the term adherence is preferred to compliance and will be used throughout the text in this paper.

In veterinary medicine, adherence is the centrepiece for fulfilling the veterinary profession's obligation to advocate on behalf of the pet's best interest. Adherence is based on effective communication of recommendations, resulting in informed client acceptance and efficient follow-through for patient care [2]. The successful outcome of a prescribed treatment depends on several factors, including a correct diagnosis, the prescription of the right treatment, and the adherence shown by the patient. The veterinarian plays a fundamental role in owner adherence to treatment of and the management of the patient, so it appears to be the result of cooperation between both persons. Since the application of any diagnostic and therapeutic option requires the owner's consent, it is of prime importance for the attending doctor to achieve their adherence to treatment, so as to achieve a successful therapeutic outcome and client satisfaction [1]. A high level of adherence in the veterinary field is dependent on two basic factors: the owner's view of his/her animal (and the resulting importance of the said animal to the owner) and the owner's understanding of the medical situation [5, 6]. The goal is to provide what the client wants, which happens to be congruent with the health care team's delivering the care the patient needs and deserves; by increasing the client's understanding of veterinary recommendations and through the health care team's reinforcement of clarifications, adherence ratios increase [2]. Adherence to treatment also appears to be influenced by the duration and frequency of the treatment, by the consultation time offered by the clinician and by the quality of interaction between the veterinarian, the pet, and the owner. With regard to the duration of the treatment, several studies have shown a negative correlation between the level of adherence and a long treatment period. In fact, if a long-term treatment is prescribed, nonadherence of the owner may occur over time which, in the case of prolonged administration of drugs, leads to reduced intake by the patient. However, with regard to the relationship between adherence and the frequency with which the treatment must be administered, it seems that a higher frequency of drug administration leads to reduced adherence to treatment; adherence is therefore inversely proportional to the increase of daily doses [7]. There is also a relationship between adherence and the time devoted by the veterinarian to the consultation: the level of adherence is generally higher in owners who believe that the veterinarian has devoted more time to the consultation [8]. In the same way, the therapist's ability to adequately explain the reasons underlying a given behaviour/symptom manifested by the animal may have a positive influence on the owner's adherence to the treatment [9]. One of the reasons that causes an owner not to adhere to the treatment is his/her belief that the treatment is not necessary, thus emphasising the need to provide a better explanation of the benefits that can be obtained through the treatment and its correct application [10]. In this way, the owners see themselves as an active part of the healing process or the maintenance of the animal's state of well-being, which has a clear positive effect on adherence to protocols that are often demanding in terms of time and economy. In line with the aim of enhancing adherence levels, the veterinarian should provide maximum clarity concerning the pathology and the necessary therapeutic protocols, so that the owner can really understand the problem and the importance of applying the correct treatments. The veterinarian should then try to empathise with the owner, through an understanding of the difficulties that the owner may encounter in the application of specific therapeutic protocols [10]. Studies performed on human psychotherapy have shown how a reliable, empathic, and flexible attitude from the therapist has a positive impact on the cooperation of the patient, while a professional with an attitude perceived as rigid, aloof, tense, distracted, and insecure has a negative influence on the adherence shown by the patient [11]. Excessive lifestyle changes can have a negative influence on adherence to treatment: asking a patient to change their lifestyle in order to improve a treatment (e.g., combining proper physical exercise and diet) is more difficult than following the pharmacological treatment alone [1]. Good adherence to treatment can also be obtained by inviting the owner to regular, scheduled follow-up visits to ensure that the treatment is being implemented correctly by the owner, modifying certain aspects based on the animal's response and encouraging the owner to express any doubts about the correct application of the treatment [12]. Only a few studies on compliance in veterinary medicine have been published and, to the authors' knowledge, no study to date has compared the pet owners' adherence in different veterinary areas. This study assessed and compared the adherence levels of the owners of patients with behavioural, cardiological, urological, and oncological problems. We investigated how the owners perceive the disease of their animal and what they think about the feasibility of the treatment proposed. Finally, we have assessed the perception of the owner related to the usefulness of the treatment and the difficulties they have encountered in implementing it.

## 2. Materials and Methods 

The study was conducted on dogs that attended the clinical visit at the Behavioural, Cardiology, Urology, and Oncology Services of the Veterinary Sciences and Public Health Department of the University of Milan from November 2012 to October 2013.

The observational prospective study was comprised of an initial enrolment phase and a second follow-up monitoring phase.

*Phase 1*. Cases were chosen among dog patients presented at the veterinary clinic for specialist consultation in the different fields considered in this study. We used a convenience sampling technique as subjects were selected because of their convenient accessibility and proximity to the researcher. Enrolment coincided with the first examination and implied a written consent of the owners. The consultations were conducted by veterinarians, specialists in the field, and were of variable duration from 45 to 120 minutes. The clinical visit was composed of medical history-taking, clinical examination, classification of the pathology, explanation of the prescribed treatment and scheduled follow-up (with health checks on varying dates depending on the pathology and the observed need), and definition of clinical outcome and prognosis.

At the end of the first examination, the veterinarian carried out an initial questionnaire with the owner, composed of 6 multiple-choice questions. Each question was worded as a statement to which the respondent assigned a score expressed by means of a Likert scale, where 1 = strongly disagree; 2 = disagree; 3 = neither; 4 = agree; 5 = strongly agree.

*Phase 2*. Then, the owners were called by phone 15 days, 1 month, 3 months, and 6 months after the examination. During the telephone calls, a questionnaire was carried out which included, in addition to the initial 6 questions, further 9 follow-up questions (Table 1).

## 3. Data Analysis

The answers to the questionnaire were classified with scores from 1 to 5 and entered into a database for later statistical analysis. The data analysis was performed by means of IBM SPSS Statistics 22 software [13]. The data was subject to a descriptive analysis and then a Chi Square test to compare the observed and expected frequencies in each response category.

## 4. Results 

This study analysed a total of 48 cases (26 spayed females, 2 intact females, 3 neutered males, and 17 intact males, with different ages ranging from 8 months to 14 years old), including 20 in the behavioural medicine area, 14 in cardiology, 8 in urology, and 6 in oncology. Some owners decided to terminate their involvement in the study (8.6%), some animals died (8.5%), and others were given away (3%) or euthanized (0.8%) (Table 2).

*Phase 1*. The analysis of the questionnaires gathered during the first examination showed how most owners (60.6%) proved to be concerned by their animal's disease (agree 33.5% and strongly agree 27.1%). Nevertheless, 42.2% of owners believe that the disease does not harm the animal's quality of life (strongly disagree 32.8%, disagree 9.4%) and are not thinking of giving it away or euthanizing it as a result of the pathology (70.3% strongly disagree). Of those interviewed, 40.4% believe that their day-to-day habits have not changed (strongly disagree 37.7%, disagree 7.6%) as a result of the animal's pathology and 53.5% find it easy to apply the new management rules recommended by the veterinarian (50.8% strongly disagree, 2.7% disagree).

These percentages vary depending on the area analysed.  Table 3, in which the “strongly agree” responses are aggregated with the “agree” responses and “disagree” is aggregated with “strongly disagree,” details the results obtained in the four areas. Owners of urological patients were the most worried about the disorder of their dogs; furthermore they did not consider abandonment or euthanasia as an option or a solution for their animals' problems.

Daily routine was more affected in owners in cardiological and behavioral areas. In addition, owners in the latter category found it more difficult than others to apply the new management rules and many of them were convinced that the disease could compromise the quality of life of their animal (Table 3).

**Figure 1:**
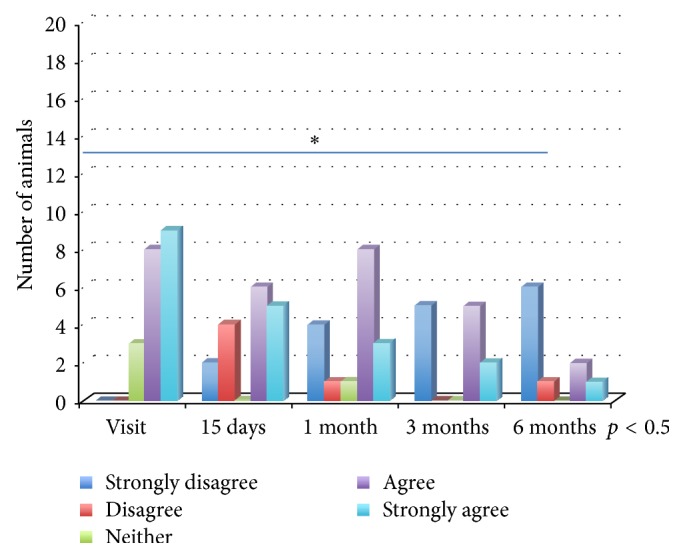
Behavioural medicine: “I am concerned about the disorder of my animal.”

**Figure 2:**
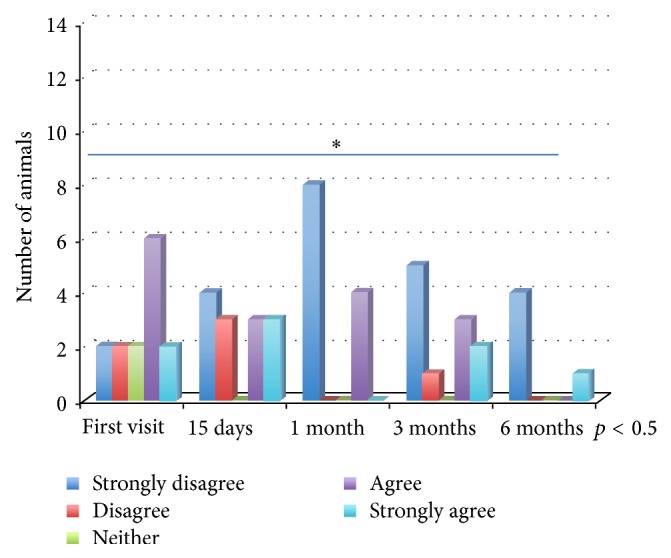
Cardiology: “My animal's quality of life is compromised by its disorder.”

*Phase 2*. The changes in the responses given by owners over time showed that, in relation to the behavioural medicine area, the concern caused by the animal's pathology significantly falls during the follow-up visits (*p* < 0.05) (Figure 1). On the contrary, the percentage of owners who consider giving their dog away as a result of the pathology increases significantly over time (*p* < 0.05). The responses to any changes in day-to-day habits and the commitment to the new management rules remain constant throughout the entire duration of the study, as is also the case with owner's responses in the cardiology and urology areas. In cardiology, the number of owners who think that the pathology harms their animal's quality of life falls significantly over time (*p* < 0.05) (Figure 2). In the oncology area, although there are a higher number of owners who, during the follow-up visits, consider the possibility of euthanizing the animal, this variation is not significant.

With regard to the 9 additional questions in the follow-up questionnaires, the only significant data relates to the behavioural medicine and urology areas, in which the owner's discomfort resulting from the animal's pathology significantly decreases over time (*p* < 0.05). In the behavioural medicine area, the discomfort caused to cohabitants and neighbours also significantly decreases (*p* < 0.05).

## 5. Discussion

The aim of our study was to assess the adherence levels of owners of patients in the behavioural, cardiological, urological, and oncological sectors of veterinary medicine. Given the lack of published research on pet owners' adherence in different veterinary areas, this pilot study was planned as a first step to determine if and how it is possible to assess and compare adherence in each of the considered sectors of veterinary medicine. The main limitation of this study is the relatively small sample of animals included, and this means that caution should therefore be exercised so as not to generalize these results and more patients should be involved in order to transfer the results to the entire population.

Overall, the owners who participated in the study appeared to be concerned by their animal's disorder; this information could be very important in improving the owner's adherence, because the concern for the pathology could represent a concrete reason for the person to adhere to the prescribed treatment [1]. However, the owner's concern does not appear to go hand in hand with the harm to the animal's quality of life. In fact, most interviewees believe that the pathology does not affect the animal's quality of life. This could be linked to poor knowledge and an erroneous interpretation of the animal's body language by the owner, which can make it difficult to recognise signs of the animal's discomfort or pain. In fact, owners often have an anthropomorphic view of their animal's behaviour and, consequently, they expect human behaviours and reactions from their animals, often creating misunderstandings in the communication and relationship with the dog [14]. Giving the animal away and putting it down are possibilities that are rarely considered by the owner. The man-animal bond of companionship changes considerably over the years and the new way of experiencing the relationship with the pet often translates into greater attention to its health and greater emotional involvement in living with its disease and its loss [15]. This greater involvement of the owner not only causes him/her not to feel the influence of the pathology on their own day-to-day habits or to encounter difficulties in adapting to the needs pertaining to their animal's pathology, but also represents an effective instrument that can be used to obtain greater adherence.

When comparing the four clinical areas investigated, the owners of patients with behavioural problems stated that they were less worried about the problem manifested by their animal and this concern tends to fall over time. This could be linked to the fact that, to date, behavioural medicine and behavioural problems are not well-known by owners and this may lead them to underestimate or not recognize the seriousness of their dog or cat's disorder or the necessary commitment to their treatment, thus reducing their adherence level. It would therefore be necessary for owners to receive more in depth information from the attending veterinarians related to the possibility of onset of behavioural pathologies [9]. Owners who, in the first visit, appeared to be most concerned were those of the urology area. In our study, patients in this area were mainly cats and currently, as a result of veterinary information, the owners of these animals have a certain level of awareness of the possibility of elderly cats developing renal pathologies; their concern could therefore be based on knowledge of such serious pathologies. A simple explanation of the pathology may encourage the owner to follow the therapeutic protocol with a greater level of participation and attention. In fact, it seems that one of the reasons that causes an owner not to adhere to the treatment is his/her belief that the treatment is not necessary or useless, thus underlining the importance of providing a clear explanation of the benefits that can be obtained through the treatment and its correct application [10]. In this way, the owners see themselves as an active part of the healing process or the maintenance of the animal's state of well-being, which has a clear positive effect on adherence to protocols that are often demanding in terms of time and money. In order to increase the adherence level, it seems important to ask owners to express their concerns about the health condition of the animal. This makes it possible to establish a dialogue with the owner, to make them feel free to express any concerns about the health of the animal and therefore add any important elements to the diagnostic process and the resulting treatment [16].

Among the four areas investigated, the owners who, upon the first visit, least consider the idea of putting down or giving the animal away as a result of the pathology are those in the urology area, followed by those of cardiology, oncology, and behavioural medicine. During the follow-up visits, the situation remains unchanged for the first three areas (in which no subject was given away or euthanized), while, in the behavioural area, four animals were given away and one was put down. For this purpose, it is important to consider the social aspect of behavioural pathology; behavioural problems often have a strong impact on the animal itself, the owner, and their cohabitants and neighbours, and this is why an owner could be more willing to give away or put down their animal [17]. Furthermore, owners often do not have sufficient information to manage the behavioural problem, they are not referred to specific, specialised professionals, and they attend specialist consultations when the problem has already been apparent for some time (thus compromising the prognosis [17, 18]). In addition to this, this factor could be influenced by the effect of time on the owner-dog relationship. In fact, in most cases, heart, urological, and oncological pathologies occur at an advanced age, that is to say, after years of close cohabitation between the person and the animal, with the resulting creation of a strong and deep-rooted bond; conversely, behavioural pathologies often arise in young animals or those recently adopted and, sometimes, this brief period is not sufficient to create a close and deep relationship. The increase in owners of patients in behavioural medicine who have given their animal away by the third follow-up can be traced back to the fact that, at this point in the treatment, the owner expects results that do not materialise because, in most cases, behavioural treatment requires time and commitment and results may not be visible after only one month from the start [18]. In this regard, it seems that the veterinarian can help to increase adherence levels by also inviting the owner to regular, scheduled follow-up visits: this would enable the veterinarian to ensure that the treatment is being implemented correctly by the owner and possibly change any aspects depending on the animal's response [1, 19].

When comparing the four areas investigated, the management rules laid down by the behavioural veterinary doctor as well as the day-to-day habits appear to be the most demanding. This may be derived from the fact that behavioural treatment, unlike that which is generally prescribed by specialists in the other three areas, is comprised of changes to the rules of management and interaction with the animal, in addition to administration of a pharmacological treatment where necessary [19]. Since the ease of applying the treatment has a positive effect on adherence levels, it is advisable to limit the amount of information provided, use clear and plain language, and speak slowly to enable the owner to absorb each indication given [2, 9, 20, 21]. Clear and simple explanations often give rise not only to greater adherence to the treatment, but also to an increase in the value attributed by the owner to the veterinary recommendations [10]. In fact, adherence to treatment appears to be significantly influenced by the importance and value given by the owner to the veterinary recommendations [10].

When comparing the four areas investigated, there are a greater number of owners in the behavioural medicine and cardiology groups who believe that the pathology harms the animal's quality of life. In the case of cardiology, there is a significant reduction of this value over time and this points to the effectiveness of the treatment enabling the owner to take into account the clinical improvements of their animal. This consideration is also reinforced by the fact that, in the same area, there is a significant decrease in the discomfort caused to the owner during the follow-up visits. The same information has also been obtained in the behavioural medicine area, in which there is also a reduction in the discomfort caused to the owner by the pathology [18].

## 6. Conclusions

In the veterinary field, the management of the patient is the result of cooperation between the veterinarian and the owner. The role of the veterinarian is therefore fundamental in enabling an increase in the levels of adherence to the therapeutic protocols by the owners of animals.

To achieve good adherence levels, constant contact between the doctor and owner is necessary. It is therefore essential that the owner be allowed to clarify any doubts with the veterinarian that may arise during treatment and to receive constant support in the administration of the treatment and coping with the animal's pathology. Assessment of adherence appears to be an optimal instrument in identifying the positive factors and the difficulties encountered by owners during the application of a treatment requiring the administration of specific drugs and the implementation of precise management rules. The results set out here should be considered as preliminary to research that we are conducting on a wider sample of animals for a longer follow-up period.

## Figures and Tables

**Table 1:** Questionnaire: 1 = strongly disagree; 2 = disagree; 3 = neither; 4 = agree; and 5 = strongly agree.

Follow-up						
	Questionnaire	1	2	3	4	5
First visit	(1) I am concerned about the disorder of my animal					
	(2) I could abandon my animal because of the disorder					
	(3) I could euthanize my animal because of the disorder					
	(4) My daily routine have changed because of the disorder of my animal					
	(5) It is challenging to apply the new management rules					
	(6) My animal quality of life is compromised by its disorder					

	(7) The disorder of my animal has been explained in detail					
	(8) The disorder of my animal bothers me					
	(9) The disorder of my animal bothers my neighbors or roommates					
	(10) The pharmacological treatment has been explained in detail					
	(11) It is simple to follow pharmacological recommendation					
	(12) I am consistent in administering drugs					
	(13) It is useful to administer prescribed drugs					
	(14) My animal refuses to take drugs					
	(15) The new management rules has been explained in detail					

**Table 2:** Number of owners who abandoned the study and number of patients abandoned, euthanized, and dead (BM = behavioural medicine; C = cardiology; U = urology; and O = oncology).

	First visit				15 days				1 month				3 months				6 months				Total			
	BM	C	U	O	BM	C	U	O	BM	C	U	O	BM	C	U	O	BM	C	U	O	BM	C	U	O
Owners who abandoned the study	0	0	0	0	2	1	0	0	0	0	0	0	2	0	0	0	0	0	0	0	**4**	**1**	**0**	**0**
Patients abandoned	0	0	0	0	0	0	0	0	0	0	0	0	3	0	0	0	1	0	0	0	**4**	**0**	**0**	**0**
Patients euthanized	0	0	0	0	0	0	0	0	0	0	0	0	1	0	0	0	0	0	0	0	**1**	**0**	**0**	**0**
Patients dead	0	0	0	0	1	0	0	1	0	1	0	0	0	1	1	0	0	3	0	3	**1**	**5**	**1**	**4**

**Table 3:** Results obtained by the four fields during the first visit.

	Behavioural medicine		Cardiology		Urology		Oncology	
	Agree	Disagree	Agree	Disagree	Agree	Disagree	Agree	Disagree
I am concerned about the disorder of my animal	49%	26%	71,2%	9,1%	77,5%	15,5%	53,3%	21%
I could abandon my animal because of the disorder	9%	61%	4,5%	75,8%	2,5%	90%	0%	76,7%
I could euthanize my animal because of the disorder	1%	74%	6%	71,2%	2,5%	82,5%	23,3%	50%
My daily routine have changed because of the disorder of my animal	37%	36%	37,9%	42,4%	22,5%	67,5%	20%	53,3%
It is challenging to apply the new management rules	32,9%	36,7%	15,4%	62,5%	9,4%	75%	4,2%	62,5%
My animal quality of life is compromised by its disorder	41,4%	29,3%	36,5%	42,4%	17,5%	70%	16,6%	43,6%
